# SARS‐CoV‐2 Reverse Zoonosis Among Cats in China: A One Health Investigation

**DOI:** 10.1111/irv.13306

**Published:** 2024-05-02

**Authors:** Sajid Umar, Shaban Muhammad, Di Gao, Pu Chen

**Affiliations:** ^1^ Global Health Research Center (GHRC) Duke Kunshan University Suzhou China; ^2^ Division of Natural and Applied Sciences (DNAS) Duke Kunshan University Suzhou China; ^3^ MSD Animal Health Shanghai Shanghai China

Dear Editor,

The interconnections between humans, animals, and the environment are often referred to as the “One Health” concept. These connections are complex and closely intertwined. During the last two decades, cat population has expanded rapidly worldwide including China. There are approximately 65 million cats in China, indicating that cats have become one of the most popular pets. This proliferation of domestic cats can lead to more human–cat interactions and heightens the risk of pathogen transmission. The intimacy of the relationships as many cat owners sleep with their cats, cuddle with them, and sometimes share food, increases the potential for disease transmission [1, 2].

A zoonosis (plural zoonoses) refers to the transmission of pathogens or diseases from animals to humans. The risk of emerging and re‐emerging zoonotic diseases is increasing due to globalization, industrialization, deforestation, climate change, and growth in international travel and trade, raising fear of new pandemics [2, 3]. One the other hand, the term reverse zoonosis or zooanthroponosis or anthroponosis specifically refers to human‐to‐animal transmission of pathogens [1, 3, 4]. Reverse zoonosis has been neglected and received far less attention in the past as compared to zoonotic events. There could be several reasons for this imbalance: First, diseases that directly affect human populations were emphasized over animal health and research funding is often directed towards human health issues. Second, zoonosis received more attention and resources over the years because of greater impact on human health and economy (SARS, MERS, SARS‐CoV‐2, H1N1, Ebola). Third, it is often hard to detect and diagnose illness in animals than humans because less systemic funds for animal disease surveillance. Furthermore, the concept of One Health is particularly important in densely populated countries like China. Pathogen spillovers from animal to humans and vice versa can have more significant public health implications due to the potential for faster spread in densely populated countries. The One Health concept has been gaining traction that acknowledges that human and animal are closely linked, and pathogen can move in both directions (Figure 1). This approach may stimulate more research into reverse zoonoses. In the last two decades, reverse zoonosis has gained attention of researchers because of zoonotic disease outbreaks (H5N1, H1N1, SARS, MERS, SARS‐CoV‐2) seriously impacting human health and economy [5]. Fagre et al. [6] documented that several pathogens including virus and bacteria are being passed from people to wild animals and reverse zoonosis events are happening continuously; however, we are not picking them up largely due to less robust sampling and veterinary diagnostic tools. Recently, viral genomic analysis revealed that humans transmit more pathogens to animals than animal transmit to humans. Furthermore, human pathogens could adapt and evolve within animal reservoirs, and hence, reverse zoonotic events not only harm animals but their spillbacks with potentially more virulent form could also reinfect human [7]. A better understanding of reverse zoonoses with One Health approach is critical to predict and mitigate future pandemic threats.

**FIGURE 1:**
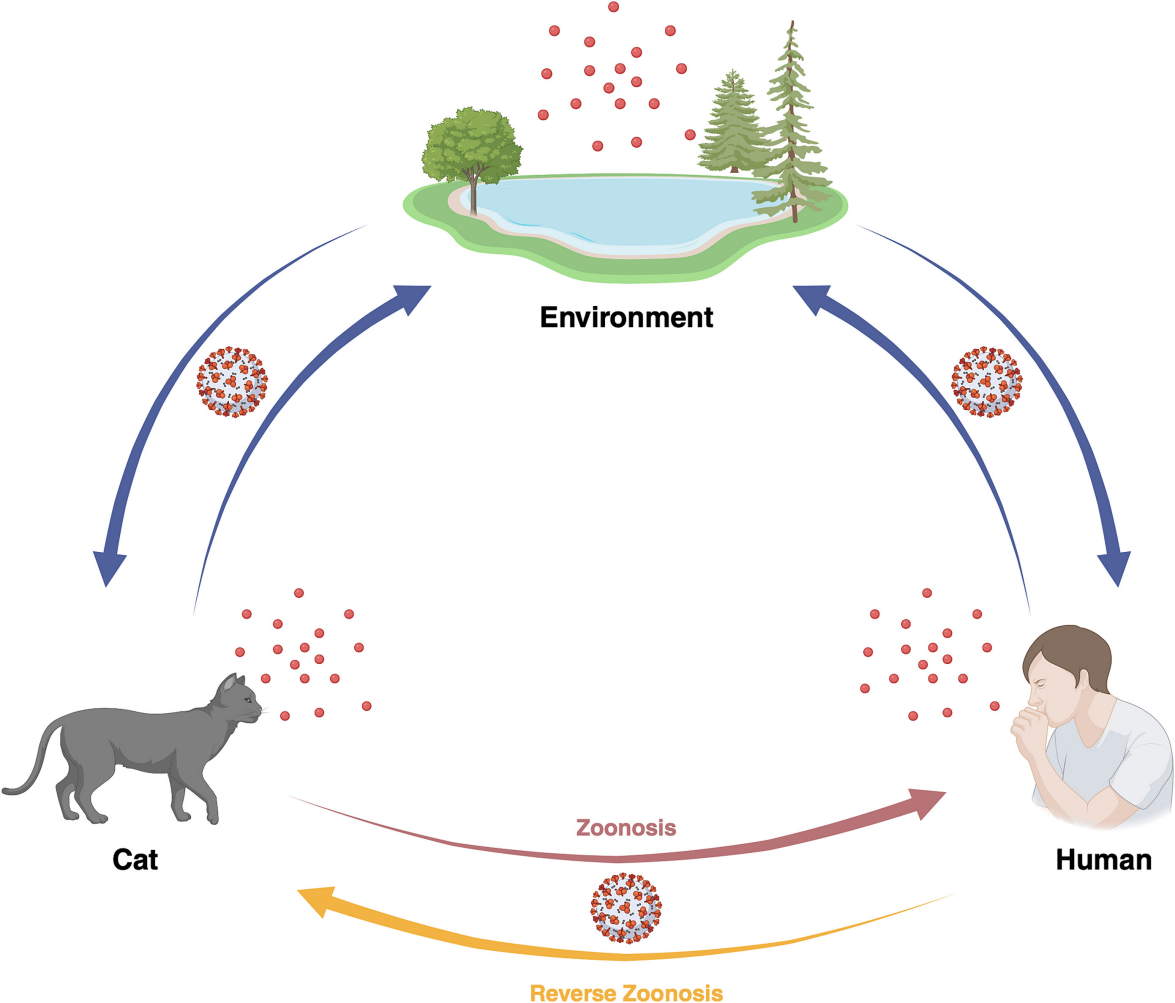
Zoonotic and reverse zoonotic events of SARS‐CoV2 among cat, human, and environment (www.biorender.com 2024).

SARS‐CoV‐2 is RNA virus within family *Coronaviridae*, which has a higher mutation rate because the enzymes that replicate its RNA genome are prone to errors and lack proofreading mechanisms. Several studies support that SARS CoV‐2 had a zoonotic origin and wildlife farming, and the wildlife trade could have spilled it to humans. SARS‐CoV‐2 infects multiple animal species, including domestic and wildlife [3, 8, 9]. Reverse zoonosis is the main cause of animal SARS‐CoV‐2 infections. Cross‐species transmission increased the spread and evolution of SARS‐CoV‐2. Dogs are less susceptible to SARS CoV‐2 infection than cats. This lower susceptibility of dogs could be due to low level of angiotensin converting enzyme 2 (ACE2) receptors and a single nucleotide mutation in dogs. The increasing global population of human and cats provides more opportunities for interaction and cross‐species transmission of SARS‐CoV‐2.

There is scarcity of data on reverse zoonosis of SARS‐CoV‐2 in China, especially in cats. During 2021–2024, a total of 458 nasal swab samples was collected from cats at veterinary clinics to monitor reverse zoonotic transmission of SARS‐CoV‐2 among cats in Kunshan city of Jiangsu Province in China. World Organization for Animal Health guidelines were strictly followed during sample collection. Samples were submerged into 5‐mL sterile virus transport media (Copan Diagnostics Inc, Italy). Takara MiniBest viral RNA extraction Kits (Cat#9766, Takara, Dalian, China) was used to extract RNA from samples. We employed one‐step real‐time RT‐PCR (RT‐qPCR) to detect SARS‐CoV‐2 within sample using MIC Real Time qPCR cycler (Biomolecular systems, Australia). SARS‐CoV‐2 was detected by following the protocols described previously [10] with Superscript III One‐Step RT‐PCR System with platinum Taq polymerase (Thermo Fisher Scientific Inc). Positive and negative controls were included in each reaction to verify our diagnostic assays. A cycle threshold (Ct) below 40 (< 40) was considered positive. In addition, a conventional RT‐PCR was employed on all SARS‐CoV‐2–positive samples to amplify partial spike (S) gene (748 bp) for sequencing [11].

This study reveals a SARS‐CoV‐2 positivity rate of 1.5% among infected cats (7/458) during this study which is lower than that reported from South Korea and other countries [9, 12]. In another study, no SARS‐CoV2 coinfection was noticed in cat samples [10], which might be partly due to the relatively lower prevalence of SARS‐CoV‐2 or could be a function of the sample size and the populations sampled. Genetic analysis of partial S gene revealed the presence of Omicron variant BA.5.2 in all positive samples from infected cats. All SARS‐CoV‐2–positive cats were sampled 2023 and 2024, which could be related to peak season of SARS CoV2 during 2023 in China. It is reported that risk of reverse zoonosis with SARS‐CoV‐2 increases with prolonged contact times between cats and cat owners [12]. Moreover, two SARS‐CoV‐2–positive samples were coinfected with feline calicivirus in this study. Surprisingly, no SARS CoV‐2 was detected in adult cats (> 4 years of age). The nondetection of SARS‐CoV‐2 in adult cats may point towards an age‐related defense mechanism or differing behavioral patterns reducing exposure risk. Interestingly, a higher detection rate (6/7, 85.7%) was observed in samples collected during cold seasons which could be linked to peak of SARS‐CoV‐2 outbreaks in Jiangsu province of China. Four cats were asymptomatic while three cats showed mild respiratory distress and sneezing. SARS‐CoV‐2–infected cats may remain asymptomatic or display signs of respiratory disease clinically indistinguishable from other respiratory pathogens [9]. All infected cats recovered well, and no death was reported. Previously, it has been reported that dogs, cats, and many other animal species were infected with SARS‐CoV‐2 during COVID‐19 pandemic [3, 8, 9, 10, 12]. Cats are susceptible to SARS‐CoV‐2 following both experimental and natural infection, although natural cases have been only sporadically reported [9]. Reverse zoonotic events remained understudied in China. Reverse zoonosis of SARS‐CoV‐2 was observed among cats in this study. Our study limited to a small geographical area within China and these SARS‐CoV‐2 zoonotic events are probably just the “tip of the iceberg.” Even with limitations, this study highlights the vulnerability of cat populations to SARS‐CoV‐2 transmission from humans and could provide useful information for veterinary public health researchers. Cats could serve as novel reservoir for SARS‐CoV‐2 and could potentially lead to cross species transmission, human reinfection, and SARS‐CoV‐2 evolution. The spillover of SARS‐CoV‐2 from humans to cats may represent a new animal reservoir with spillback potential to humans. In addition, SARS‐CoV‐2 infected cats could become a potential threat to endangered wildlife species and biodiversity conservation. This serious concern with reverse zoonosis of SARS‐CoV‐2 could potentially complicate efforts to control the pandemic and might lead to the emergence of vaccine‐resistant strains.

Despite susceptibility of cats to natural and experimental SARS‐CoV‐2 infections, the role of SARS‐CoV‐2 in the development of feline respiratory disease complex is not established yet. However, screening for SARS‐CoV‐2 during feline respiratory disease complex could be relevant from human reinfection standpoint [10]. Since cat‐to‐human transmission has already been reported [5, 8], therefore, vaccination of cats against SARS‐CoV‐2 should be considered along with animal owners as additional measure. Vaccination could reduce virus replication and host to host transmission of SARS‐CoV‐2. We suggest that targeted surveillance studies should be initiated along with precautionary measures to mitigate the impact of SARS‐CoV‐2 reinfections. Close contact between owners and the cats likely led to the transmission, emphasizing the crucial need for strict infection control measures during the period of SARS CoV‐2 infections in animal owners. We can reduce the risk of transmission by frequent handwashing especially before and after handling cats, their beddings, water, and food supplies. SARS CoV‐2 infected persons are advised to limit contacts with cats as much as possible including cat kisses and close snuggles. Wearing protective clothing and keeping the environment clean could minimize spread of SARS‐CoV‐2 to their cats. Cat could serve as a new animal reservoir for SARS‐CoV‐2 and could be a potential threat to public health. Therefore, a holistic One Health approach is highly desired for continuous epidemiological surveillance and monitoring the emergence of new potential variants of SARS‐CoV‐2 among cats and humans.

## Author Contributions

**Sajid Umar:** Conceptualization; Investigation; Funding acquisition; Methodology; Validation; Visualization; Writing – review and editing; Writing – original draft; Formal analysis; Supervision; Project administration. **Shaban Muhammad:** Investigation; Writing – review and editing; Writing – original draft. **Di Gao:** Investigation; Writing – review and editing; Writing – original draft. **Pu Chen:** Investigation; Writing – original draft; Writing – review and editing.

## Conflicts of Interest

The authors declare no conflicts of interest.

### Peer Review

The peer review history for this article is available at https://www.webofscience.com/api/gateway/wos/peer‐review/10.1111/irv.13306.

## Data Availability

The data that support the findings of this study are available on request from the corresponding author. The data are not publicly available because of privacy or ethical restrictions.
